# Takotsubo cardiomyopathy in a snake bite victim: a case report

**Published:** 2012-11-15

**Authors:** Kichiro Murase, Kenji Takagi

**Affiliations:** 1 Nagoya University School of Medicine,Nagoya, Japan; 2 Department of general surgery, Sakashita Hospital, Japan

**Keywords:** Snake bite, Takotsubo cardiomyopathy

## Abstract

Takotsubo cardiomyopathy occurs in patients with severe emotional or physiologic stress. The prognosis is usually favorable, and the left ventricular wall motion dyskinesis normalizes within days to weeks. In this paper we report a case of snake bite complicated by takotsubo cardiomyopathy. We advise physicians to consider not only cardioinhibitory effect of snake venom but also takotsubo cardiomyopathy as a differential diagnosis if the patient develops cardiac dysfunction following snake bite.

## Introduction

Takotsubo cardiomyopathy, also called stress-induced cardiomyopathy or transient apical ballooning, has been described as a novel cardiac syndrome of left ventricular apical ballooning, electrocardiographic changes, and minimal elevation of cardiac enzymes resembling acute myocardial infarction, but without evidence of coronary artery disease [1, 2]. Characteristic left ventricular apical ballooning in end-systole resembles a pot with round bottom and narrow neck used as an octopus trap called “Takotsubo” in Japanese. Takotsubo cardiomyopathy predominantly occurs in postmenopausal women, and a period of acute emotional or physiologic stress precedes the onset of symptoms in most cases. The prognosis is usually favorable, and the left ventricular wall motion dyskinesis normalizes within days to weeks. Here we report a case of Takotsubo cardiomyopathy associated with snake bite.

## Patient and observation

A 56-year-old female was bitten by a snake on her left foot. Soon after, she was taken to our hospital. The patient was conscious and without any neurological deficits. Her vital signs were stable on arrival and during in hospital period. Her left foot showed swelling, which gradually worsened over the next few days and progressed to the left thigh.

The laboratory exams showed elevations of creatine kinase (maximum 37340 IU/L on day 3) and white cell count (maximum 14520/ micro L on day 2). Creatine kinase-MB fraction (4%) remained within normal range.

Antibiotics and tetanus vaccine as well as intravenous fluid were administered. Anti snake venom was not administered. Cepharanthine was administered intravenously. Cepharanthine is a biscoclaurine (bisbenzylisoquinoline) amphipathic alkaloid isolated from *Stephania cepharantha* Hayata and is widely used in Japan to treat a variety of diseases including alopecia areata, radiotherapy-induced leucopenia, and, septic shock [3].

**Figure 1:**
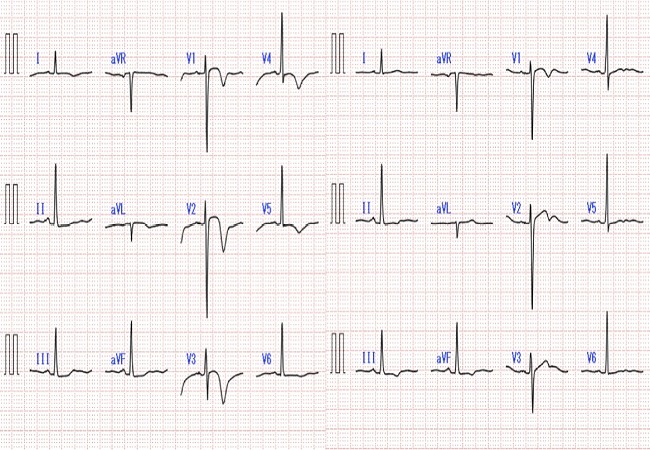
Electrocardiogram on admission (left) and four days after admission (right). T wave inversion observed on admission resolved after four days

The electrocardiogram, which is routinely recorded in admitted patient in our hospital, revealed negative T waves in various leads (Figure 1). Therefore we undertook echocardiogram and observed abnormal left ventricular systolic function with apical akinesis and a hyperdynamic basal segment of the left ventricle, which is typical in Takotsubo cardiomyopathy. Abnormalities in electrocardiogram and echocardiogram resolved four days after snake bite. Hemodynamic complications including heart failure and severe arrhythmia did not occur. Multi-detector CT scanning undertaken later on revealed no stenotic lesions in her coronary arteries.

She was discharged without any disabilities after two months of rehabilitation.

The victim could not identify the snake, however, we assume that the snake was *Gloydius blomhoffii*, since there are only two species of venomous snake, *G. blomhoffii* and *Rhabdophis tigrinus*, in the patient's habitation, and the fang site was likely due to *G. blomhoffii*.

## Discussion

Transient cardiac dysfunction in this case can be attributed to the snake venom. However, considering rapid recovery of the patient's heart function and normal creatine kinase-MB isozyme, this is unlikely. Our case met all four of the proposed Mayo Criteria for the clinical diagnosis of transient left ventricular apical ballooning syndrome (Takotsubo cardiomyopathy) [1], therefore we diagnosed this case as Takotsubo cardiomyopathy.

This case is worth reporting since there was no report of a case of Takotsubo cardiomyopathy associated with snake bite. Snake bite is an alarming and frightening experience to the victim, so there may be more cases with transient left ventricular dysfunction. But similar cases are probably unnoticed as the physicians’ primary concern is physical recovery and echocardiography is sometimes unavailable in areas where snake bite is endemic.

The prognosis of patients with Takotsubo cardiomyopathy is generally favorable like the case presented here, however, heart failure is the most common clinical complication [1]. There are no specific treatments for Takotsubo cardiomyopathy, but supportive measures such as administering oxygen and diuretics for pulmonary edema are needed in many cases. And care should be taken to arrhythmia resulting from QT prolongation.

Our case report alerts physicians managing snake bite to consider not only cardioinhibitory effect of snake venom but also Takotsubo cardiomyopathy as a differential diagnosis if the victim developed cardiac dysfunction.

## Conclusion

A case of Takotsubo cardiomyopathy associated with snake bite is reported for the first time. Physician should bear in mind that emotional calamity and fright render the snake bite victims susceptible to Takotsubo cardiomyopathy.
